# Factors Underlying Vaccine Hesitancy and Their Mitigations in Saudi Arabia: Protocol for a Systematic Review

**DOI:** 10.2196/54680

**Published:** 2024-03-22

**Authors:** Mohammed Kanan, Samar Abdulrahman, Abdulaziz Alshehri, Renad AlSuhaibani, Nawaf M Alotaibi, Azhar Alsaleh, Bushra Nasser, Rana Baowaydhan, Ibrahim Alredaini, Taif Khalid, Fatima Almukhtar, Nourah Altoaimi, Almaha Alhneshel, Shouq Alanazi, Shahad Algmaizi

**Affiliations:** 1 Department of Clinical Pharmacy King Fahad Medical City Riyadh Saudi Arabia; 2 Department of Pharmacy Second Health Cluster Riyadh Saudi Arabia; 3 Department of Pharmacy Security Forces Hospital Riyadh Saudi Arabia; 4 Department of Medicine Imam Mohammed Ibn Saud Islamic University Riyadh Saudi Arabia; 5 Department of Clinical Pharmacy Northern Border University Rafha Saudi Arabia; 6 Department of Nursing Prince Saud Bin Jalawi Hospital Alhasa Saudi Arabia; 7 Department of Clinical Pharmacy King Khalid University Abha Saudi Arabia; 8 Department of Medicine King Abdulaziz University Jeddah Saudi Arabia; 9 Department of Pharmacy Qassim University Buraydah Saudi Arabia; 10 Department of Clinical Laboratory Al-Jawf University Al-Jawf Saudi Arabia; 11 Department of Medicine Ministry of Health Dammam Saudi Arabia; 12 Department of Pharmacy Qassim University Qassim Saudi Arabia; 13 Department of Medicine Suliman Alrajhi University Qassim Saudi Arabia; 14 Department of Medicine Northern Border University, Northern Borders Arar Saudi Arabia; 15 Department of Medicine King Saud bin Abdulaziz University for Health Sciences Riyadh Saudi Arabia

**Keywords:** acceptance, campaigns, effectiveness, factors, hesitancy, immunization rates, immunization, intervention, literature analysis, misinformation, mitigations, prevention, protocol, public health, review methodology, review methods, Saudi Arabia, search, searching, syntheses, synthesis, systematic review, systematic, vaccination, vaccine hesitancy, vaccine

## Abstract

**Background:**

Vaccine hesitancy is a growing concern in Saudi Arabia, impacting even well-educated parents. The decision-making process involves various factors such as accessibility, trustworthy information, and the influence of social networks, reflecting a complex interplay of emotional, cultural, social, spiritual, and political dimensions.

**Objective:**

This review seeks to evaluate the prevalence and trends of vaccine hesitancy, identify contributing factors, and explore potential solutions to enhance immunization rates. This review aligns with global concerns, as the World Health Organization has identified vaccine hesitancy as a top global health threat.

**Methods:**

Our systematic review will follow the PRISMA (Preferred Reporting Items for Systematic Reviews and Meta-Analyses) guidelines and PICOS (Population, Intervention, Comparison, Outcomes, and Study) criteria for comprehensive assessment. We will conduct a thorough search across various databases, encompassing a wide range of vaccines, and pay special attention to vaccination campaigns and refusals. Inclusion criteria involve descriptive, observational, and analytical studies focusing on factors influencing vaccine acceptance or hesitancy. The study will use the Crowe Critical Appraisal Tool for quality assessment and perform a narrative synthesis to summarize findings thematically.

**Results:**

This systematic review is expected to unveil the prevalence and trends of vaccine hesitancy in diverse populations in Saudi Arabia, shedding light on cultural, religious, and social factors contributing to hesitancy. It aims to assess the effectiveness of implemented strategies, enable regional and global comparisons, and provide implications for tailored vaccination policies. Additionally, the review may pinpoint research gaps, guiding future investigations to address and mitigate vaccine hesitancy effectively.

**Conclusions:**

The findings are expected to have direct policy implications and guide interventions to strengthen vaccination programs and improve public health outcomes.

**International Registered Report Identifier (IRRID):**

PRR1-10.2196/54680

## Introduction

Vaccines have consistently proven to be among the safest and most effective methods for preventing a wide range of infectious diseases [1,2]. Despite the proven safety and effectiveness of vaccines, vaccine-preventable diseases continue to persist in various parts of the world. In recent years, there have been outbreaks of infectious diseases, even when effective vaccines are available to combat them. One significant contributing factor to this phenomenon is “vaccine hesitancy” [3,4]. Vaccine hesitancy is defined as a reluctance or delay in accepting or agreeing to receive vaccines, even when vaccination services are readily available. Vaccine hesitancy is a complex and multifaceted phenomenon influenced by a wide range of factors. These factors encompass cognitive, psychological, sociodemographic, political, and cultural elements, among others. Moreover, the specific factors contributing to vaccine hesitancy can vary significantly across different populations and communities [5]. The reasons for vaccine hesitancy are intricate and can vary over time, across different locations, and depending on the specific type of vaccine in question. Similarly, vaccine hesitancy arises from a multitude of factors, including religious beliefs, geographic barriers, the quality of the parent-provider relationship, concerns about adverse events of immunization, limited knowledge about vaccination, and perceptions of disease risk [6].

In 2019, the World Health Organization (WHO) identified vaccine hesitancy as one of the top 10 global health threats [7]. Vaccine hesitancy refers to the reluctance of certain individuals or communities to accept vaccines, and it poses a substantial challenge to public health efforts. This reluctance is influenced by various factors, including misinformation, distrust in health care systems, personal beliefs, and a perceived low risk of vaccine-preventable diseases [7,8]. Vaccine hesitancy is not a recent challenge in disease prevention. It has been a significant issue for years, and examples of it can be found in the context of seasonal influenza vaccination and the response to the 2009 H1N1 pandemic [9-11]. Recent research in the literature over the past decade has indicated a concerning trend: vaccine hesitancy appears to be on the rise among various populations, including health care workers [12,13]. According to Olive et al [14], a social movement opposing public health vaccines has been gaining traction in the United States. This movement, among various other factors, has played a role in the increasing percentage of the population in both the United States and Europe that is refusing vaccination efforts in recent years [14]. Vaccine hesitancy indeed poses a substantial challenge to public health experts as it leads to significantly reduced vaccination rates within populations. This challenge becomes even more critical in the context of combating infectious diseases [1]. According to a survey conducted by the WHO and UNICEF (United Nations Children’s Fund), vaccine hesitancy started to emerge as a significant concern approximately a decade ago [15]. This trend of vaccine hesitancy has been observed in several countries around the world, including the United Kingdom, the United States, and India [16,17]. Vaccine hesitancy across Gulf Cooperation Council countries varied between 11% and 71%, with notable discrepancies observed based on the type of vaccine, with the highest reported hesitancy recorded for the COVID-19 vaccine at 70.6% [18].

Vaccine hesitancy has also become a concern in Saudi Arabia. According to Alabbad et al [19], 17% of their study population expressed hesitance to receive the influenza vaccine. Additionally, Alsubaie et al [20] found that vaccine hesitancy among Saudi parents reached 20%. Even well-educated parents have the same behavior. Alzahrani and Alghamdi [21] reported a vaccine hesitancy rate 20%-27 % against COVID-19 immunization [21]. A study conducted by Thabit et al [22] determined that factors such as the convenience of receiving the vaccine, the availability of trustworthy information from authorities, and the positive influence of family and friends played significant roles in motivating the public to get vaccinated. Al-Mohaithef and Padhi [23] reported that concerns about vaccine safety (17%), worries about potential side effects (35%), and perceptions of receiving too many injections (28%) are critical factors contributing to vaccine hesitancy. Alaamri et al [24] conducted a cross-sectional study in Saudi Arabia, which stated that decision-making for vaccination is a multifaceted process that encompasses emotional, cultural, social, spiritual, and political dimensions. These various aspects can significantly influence an individual’s or a community’s choice regarding vaccination. In-depth literature analysis reveals the current absence of a systematic review on vaccine hesitancy factors specific to Saudi Arabia. This review aims to (1) assess the prevalence and trends of vaccine hesitancy, (2) identify factors contributing to vaccine hesitancy, and (3) explore potential solutions to enhance immunization rates.

These findings will be instrumental in shaping a comprehensive vaccination policy for Saudi Arabia.

## Methods

### Basic Strategy

This systematic review will adhere to the quality standards outlined in the PRISMA (Preferred Reporting Items for Systematic Reviews and Meta-Analyses) reporting guidelines [25,26] and will be structured according to the PICOS (Population, Intervention, Comparison, Outcomes, and Study) criteria for comprehensive and transparent reporting [27], as given in Textbox 1 and Table 1. Before conducting this review, the protocol will be submitted to PROSPERO (the International Prospective Register of Systematic Reviews) for approval [28,29].

A comprehensive search will be conducted across multiple databases, including Cochrane, PubMed (MEDLINE), Web of Science, Google Scholar, Scopus, Science Direct, and Springer Links, to identify peer-reviewed literature. The search will not be limited by a specific time period because no previous review on the factor has been conducted. If it is time-limited, there is a chance of missing some factor that may affect the prospective work. It will also encompass literature with search terms present in the title, abstract, and full text. Furthermore, the search will be restricted to publications available in the English language.

**Title and checklist items for the PRISMA (Preferred Reporting Items for Systematic Reviews and Meta-Analyses) checklist.**

**Title**

- Determine whether the report is categorized as a systematic review, a meta-analysis, or a combination of both.

**Background: objectives**

- The research inquiry encompasses elements such as comparators, interventions, outcomes, and participants.

**Methods: eligibility criteria**

- Criteria for inclusion are based on the characteristics of the study and the report.

**Methods: sources of information**

- The databases that were searched and the dates of those searches.

**Methods: potential for bias**

- Methods for evaluating the potential for bias in research studies.

**Results: studies inclusion**

- The quantity and nature of incorporated research studies, the participants involved, and pertinent attributes of these studies.

**Results: report of findings**

- Summary of primary results, ideally with a breakdown of the number of studies and participants for each outcome.

**Results: description of the effect**

- The direction of the impact (ie, which group benefits) and the magnitude of the effect expressed in terms that are meaningful to health care professionals and patients.

**Discussion: limitations and strengths of available evidences**

- Concise overview of the advantages and drawbacks of the evidence, including factors like inconsistency, imprecision, indirectness, or risk of bias, along with any corroborating or conflicting evidence.

**Discussion: interpretation**

- Overall interpretation of the findings and significant implications.

**Table 1:** PICOS (Population, Intervention, Comparison, Outcomes, and Study) inclusion and exclusion criteria.

PICOS criteria	Inclusion criteria	Exclusion criteria
Participants	The community of Saudi Arabia	Research studies will intentionally exclude certain subgroups, such as those with comorbidities, complex patients, or health care providers
Intervention	The qualitative and quantitative studies and randomized controlled trials	Studies pertaining to other topics and those involving statistical, mathematical, or predictive methods will not be covered in this context
Comparison	When applicable, this comparison of specific subgroups may also extend to studies conducted in multiple cities or locations	N/A^a^
Outcomes	To assess the factors that influence individuals’ decisions regarding vaccine acceptance or vaccine hesitancy	Studies that do not aim to identify or analyze the factors influencing vaccine acceptance or hesitancy
Study design	Observational or descriptive and analytical studies conducted at a national level, or those with an adequately justified sample size and calculations	Pilot studies for tool assessment or improper sampling

^a^N/A: Not applicable.

### Searching Approaches

To optimize search results and ensure comprehensive coverage, a combination of medical subject headings and natural language keywords will be used. Boolean and proximity operators will also be used to refine search queries and uncover relevant studies. Additionally, truncation (*) and wildcards ($) will be used to account for variations in search terms and enhance the chances of identifying a wide range of relevant literature.

The search strategy has been configured to align with the specific vocabularies and indexing systems used by each database, ensuring that the search is optimized for each platform’s unique structure and content. This approach enhances the precision and relevance of search results within each database.

The search strategy will be formulated using the vocabularies and indexing systems specific to each database. This tailored approach ensures that the search is optimized for the unique characteristics and content of each database. The terms “vaccine,” “immunization,” “vaccine hesitancy,” “vaccine trust,” “vaccine resistance,” “vaccine concern,” “vaccination,” “vaccine intervention,” “vaccine side effects,” “vaccine confidence,” “vaccine impact,” “vaccine strategy,” “vaccine hesitant,” “vaccine refusal,” “adverse effect of vaccines,” and “vaccine rejection” will be applied alone or combined as operators.

### Eligibility Criteria

While conducting our search for vaccine hesitancy, we will take into account universally recommended vaccines for individuals across various age groups, including children, adolescents, and adults. These vaccines encompass a range of preventable diseases and include: “seasonal influenza vaccine,” “hepatitis B vaccine,” “tuberculosis (Bacillus Calmette-Guerin [BCG] vaccine),” “measles, mumps, and rubella (MMR) vaccine,” “diphtheria-pertussis-tetanus (DPT) vaccine,” “Haemophilus influenzae type b (Hib) vaccine,” “human papillomavirus (HPV) vaccine,” “poliomyelitis vaccine,” “oral polio vaccine,” “varicella vaccine,” “meningococcal vaccine,” “pneumococcal vaccine,” and “COVID-19 vaccine.” These vaccines are typically recommended as part of immunization programs to protect individuals from various infectious diseases. In addition, we will pay particular attention to the results of vaccination campaigns aimed at promoting and expanding vaccination programs in Saudi Arabia. This will also involve a detailed examination of the various campaigns, their objectives, strategies, and outcomes, as well as their impact on increasing vaccine coverage and public health in the region.

In this review, we will include both descriptive and analytical studies that provide insights into the impact of strategies aimed at addressing vaccine hesitancy. These studies should offer a clear description of the strategies used and their effects on vaccine hesitancy.

However, we will exclude studies that are opinion-based or studies that do not primarily focus on populations eligible to receive vaccines or their parents. Additionally, studies that do not allow for the extraction of relevant information related to vaccination will also be excluded from our analysis.

### Selection of Studies and Critical Appraisal

Our research review process should be robust, and it should be designed to ensure the quality and relevance of the studies included in the analysis [30]. The following sections provide a summary of the key steps.

#### Initial Screening

A total of 2 researchers will independently review the titles, abstracts, and keywords of the identified studies. This step will ensure the segregation of eligible studies from those that are not relevant.

#### Full-Text Retrieval and Screening

After the initial screening, studies that pass this stage will have their full texts retrieved and reviewed. This allows for a more detailed assessment of their eligibility.

#### Data Extraction

A total of 2 researchers will independently perform data extraction from the selected studies. This process involves extracting relevant information and data from the studies.

#### Consensus on Unmatched Studies

In cases where there is a disagreement between the 2 researchers regarding the inclusion or exclusion of a study, a third researcher will be involved to reach a consensus. This ensures that the final selection of studies is based on consensus and reduces bias.

This screening process, along with independent data extraction and consensus resolution, will help to enhance the reliability and validity of our research findings. It also minimizes the potential for bias in the selection and extraction of data from the identified studies.

#### Quality Assessment

This study will undergo quality assessment using the Crowe Critical Appraisal Tool (CCAT) [31,32]. This tool focuses on 8 domains for evaluating quality, encompassing preliminaries (title, abstract, and text), introduction (background and objective), design (research design, intervention, treatment, exposure, outcome, output, predictor, measure, and bias), sampling (sampling method, sample size, sampling protocol), data collection (collection method, collection protocol), ethical considerations (participant ethics, researcher ethics), results (analysis, integration, interpretation method, essential analysis, outcome, output, predictor analysis), and discussion (interpretation, generalization, and concluding remarks). The CCAT tool will be used to evaluate the quality of all the included articles across these 8 domains. A cumulative score will be computed for each article and then converted into a percentage. The percentage scores will be categorized into three groups: (1) high quality (≥80%), (2) medium quality (60%-79%), and (3) poor quality (<60%). This assessment of quality will be carried out independently by the authors, with any disagreements being resolved through consensus.

### Analysis and Synthesis

According to the methodological nature of our systematic review, conducting a narrative synthesis of the results will be a suitable approach. A narrative synthesis will involve summarizing and interpreting the findings of the included studies. This approach is common when the included studies are diverse in terms of methodology, outcome measures, or data presentation [33].

#### Thematic Analysis

We will organize the findings thematically. Focus on the common themes, patterns, or trends across the studies. We will create categories, themes, and subthemes that capture the key aspects of vaccine hesitancy and the impact of strategies.

#### Narrative Description and Synthesis

We will describe the findings of each study within the context of the identified themes that provide a clear and concise summary of each study. We will synthesize the findings by drawing connections between different studies and themes.

## Results

We can anticipate potential results that may emerge from this systematic review.

### Prevalence and Trends of Vaccine Hesitancy

The review is expected to reveal the prevalence and trends of vaccine hesitancy among different populations. It may show whether vaccine hesitancy rates have been increasing or decreasing over time and how this compares to global trends.

### Factors Contributing to Vaccine Hesitancy

Identification of specific factors contributing to vaccine hesitancy in the Saudi Arabian context. Insights into cultural, religious, and social influences on vaccine hesitancy within Saudi Arabia.

### Impact of Strategies on Vaccine Hesitancy

Evaluation of the effectiveness of strategies implemented to address vaccine hesitancy and the identification of successful interventions and areas where improvements are needed.

### Regional and Global Comparisons

Discover potential comparisons between vaccine hesitancy factors and strategies in Saudi Arabia and those in other countries, particularly within the Middle East region, and understand whether Saudi Arabia faces unique challenges or shares common issues with other nations.

### Implications for Vaccination Policy

The review is likely to have implications for public health policy in Saudi Arabia and provide recommendations for tailored vaccination strategies to improve acceptance rates and combat vaccine hesitancy.

### Identifying Research Gaps

The review may identify gaps in the existing literature on vaccine hesitancy in Saudi Arabia and highlight areas where further research is needed or not to address vaccine hesitancy.

## Discussion

### Impact

Vaccine hesitancy poses a substantial threat to public health worldwide [34], and Saudi Arabia is no exception [19-24]. Understanding the prevalence and factors contributing to vaccine hesitancy in Saudi Arabia is crucial for developing targeted interventions to increase vaccination rates and prevent outbreaks of vaccine-preventable diseases [35,36]. Moreover, the causes of vaccine hesitancy and its consequences will be helpful to mitigate [37].

Like the United States, the findings will help tailor an individualized educational program for vaccine-hesitant parents [38]. Alternatively, childhood vaccination will increase, as it is reported to be very low in Saudi Arabia [39-41]. Delays in vaccination were influenced by factors such as parental education, nutrition preferences, and vaccine-related beliefs, while prematurity was associated with a decreased likelihood of delays. As a mandatory requirement, children in Saudi Arabia are expected to be fully vaccinated before starting school at the age of 6 years [42].

In Saudi Arabia, the vaccination program commenced in 1979 with the administration of DTP vaccines, and it has since been expanded to encompass a broader range of vaccines [20]. The Saudi National Immunization Program advises administering several vaccines within the first 24 months of life. These vaccines include the hepatitis B vaccine at birth, as well as the pneumococcal conjugate, rotavirus, inactivated poliovirus vaccine (IPV), meningococcal vaccine (MCV), diphtheria, tetanus, polio, Hib vaccine, BCG vaccine, MMR vaccine, and hepatitis A vaccines [20,42].

According to the WHO, vaccination coverage rates in Saudi Arabia have shown significant variation in recent years. In 2019, the coverage rate for BCG vaccination was reported to be 52%, a notable decrease from the 98% coverage rate observed in 2018 [42,43]. While in 2022, it was similar to 2022 [44]. On the other hand, the coverage rates for DPT and MMR vaccines remained high, with rates of 98% and 96%, respectively. Despite the generally high immunization rates, there were concerning numbers of reported cases in 2019. Measles, for instance, had 1035 reported cases, mumps had 187 reported cases, pertussis had 326 reported cases, and rubella had 62 reported cases. These figures indicate that, despite high vaccination coverage rates for some vaccines, there are still significant challenges in preventing these vaccine-preventable diseases in certain populations or regions within Saudi Arabia [42,43].

The study aligns with global concerns about vaccine hesitancy, which the WHO identified as a top 10 global health threat [7]. By exploring vaccine hesitancy in Saudi Arabia, this research contributes to the global understanding of this phenomenon and may shed light on whether Saudi Arabia faces unique challenges or shares common issues with other nations [45,46].

By analyzing the predictors from different stakeholders [47-51], the study’s findings are likely to have direct policy implications. Tailored vaccination strategies can be developed based on the identified factors contributing to vaccine hesitancy [41-43,52]. Policy recommendations can help improve acceptance rates and strengthen vaccination programs in Saudi Arabia, ultimately enhancing public health outcomes. Identifying specific factors contributing to vaccine hesitancy in Saudi Arabia is essential for designing targeted interventions. For instance, if religious beliefs are a significant factor [53,54], interventions may involve engaging religious leaders to promote vaccination [55].

### Conclusions

This systematic review will investigate Saudi Arabia’s vaccine hesitancy, examining prevalence, trends, and contributing factors across diverse populations. It evaluates the effectiveness of implemented strategies and draws regional and global comparisons, shedding light on unique challenges or shared issues within the Middle East. The findings hold implications for Saudi public health policy, suggesting tailored vaccination strategies to combat hesitancy. Additionally, by identifying research gaps, the review provides direction for future studies, pinpointing areas where further investigation is crucial for a comprehensive understanding of vaccine hesitancy in Saudi Arabia.

## Abbreviations

BCG: Bacillus Calmette-Guerin
CCAT: Crowe Critical Appraisal Tool
DPT: diphtheria-pertussis-tetanus
Hib: Haemophilus influenzae type b
HPV: human papillomavirus
IPV: inactivated poliovirus vaccine
MCV: meningococcal vaccine
MMR: measles, mumps, and rubella
PICOS: Population, Intervention, Comparison, Outcomes, and Study
PRISMA: Preferred Reporting Items for Systematic Reviews and Meta-Analyses
PROSPERO: International Prospective Register of Systematic Reviews
UNICEF: United Nations Children’s Fund
WHO: World Health Organization
